# 5-Carb­oxy-2-isopropyl-1*H*-imidazol-3-ium-4-carboxyl­ate monohydrate

**DOI:** 10.1107/S1600536811024767

**Published:** 2011-06-30

**Authors:** Chao-Jun Du, Zheng-Hai Shi, Li-Sheng Wang, Chao-Ling Du

**Affiliations:** a Department of Chemical and Biochemical Engineering, Nanyang Institute of Technology, 473004 Nanyang, Henan, People’s Republic of China; b School of Chemical Engineering and Environment, Beijing Institute of Technology, 100081 Beijing, People’s Republic of China; c College of Science, Nanjing University of Aeronautics and Astronautics, Nanjing 211100, People’s Republic of China

## Abstract

In the title compound, C_8_H_10_N_2_O_4_·H_2_O, the imidazole N atom is protonated and one of the carboxyl­ate groups is deprotoned, forming a zwitterion. An intra­molecular O—H⋯O hydrogen bond occurs. The crystal structure is stabilized by inter­molecular N—H⋯O and O—H⋯O hydrogen bonds. In addition, inter­molecular N—H⋯O and O—H⋯O hydrogen bonds link the mol­ecules into two-dimensional networks parallel to (10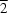).

## Related literature

For the use of related imidazole­dicarb­oxy­lic acid structures in coordination chemistry, see: Sun *et al.* (2006 ▶); Merchan & Stoeckli-Evans (2007 ▶); Guo (2009 ▶); Wang & Qin (2010 ▶); Wang *et al.* (2010 ▶); Feng *et al.* (2010 ▶); Li *et al.* (2010 ▶). For the synthesis of the title compound, see: Alcalde *et al.* (1992 ▶).
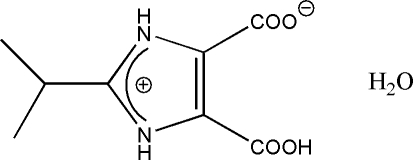


## Experimental

### 

#### Crystal data

C_8_H_10_N_2_O_4_·H_2_O
*M* *_r_* = 216.20
Monoclinic, 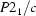

*a* = 7.828 (2) Å
*b* = 14.308 (4) Å
*c* = 8.930 (2) Å
β = 93.590 (3)°
*V* = 998.2 (4) Å^3^
*Z* = 4
Mo *K*α radiation
μ = 0.12 mm^−1^
*T* = 298 K
0.40 × 0.32 × 0.28 mm

#### Data collection

Bruker SMART APEXII CCD area-detector diffractometer
Absorption correction: multi-scan (*SADABS*; Bruker, 2005 ▶) *T*_min_ = 0.953, *T*_max_ = 0.967
4874 measured reflections
2147 independent reflections
1599 reflections with *I* > 2σ(*I*)
*R*_int_ = 0.028

#### Refinement

*R*[*F* ^2^ > 2σ(*F* ^2^)] = 0.043
*wR*(*F* ^2^) = 0.123
*S* = 1.06
2147 reflections
140 parameters
3 restraints
H-atom parameters constrained
Δρ_max_ = 0.32 e Å^−3^
Δρ_min_ = −0.19 e Å^−3^

### 

Data collection: *APEX2* (Bruker, 2005 ▶); cell refinement: *SAINT* (Bruker, 2005 ▶); data reduction: *SAINT*; program(s) used to solve structure: *SHELXTL* (Sheldrick, 2008 ▶); program(s) used to refine structure: *SHELXTL*; molecular graphics: *SHELXTL*; software used to prepare material for publication: *SHELXTL*.

## Supplementary Material

Crystal structure: contains datablock(s) global, I. DOI: 10.1107/S1600536811024767/lr2015sup1.cif

Structure factors: contains datablock(s) I. DOI: 10.1107/S1600536811024767/lr2015Isup2.hkl

Supplementary material file. DOI: 10.1107/S1600536811024767/lr2015Isup3.cml

Additional supplementary materials:  crystallographic information; 3D view; checkCIF report

## Figures and Tables

**Table 1:** Hydrogen-bond geometry (Å, °)

*D*—H⋯*A*	*D*—H	H⋯*A*	*D*⋯*A*	*D*—H⋯*A*
O3—H3⋯O2	0.82	1.64	2.4576 (19)	175
N1—H1⋯O5	0.86	1.83	2.6879 (19)	171
N2—H2⋯O1^i^	0.86	1.93	2.7619 (19)	162
O5—H5*B*⋯O3^ii^	0.85	2.03	2.8684 (19)	171
O5—H5*A*⋯O4^iii^	0.85	1.94	2.7857 (19)	173

Symmetry codes: (i) 
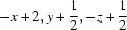
; (ii) 
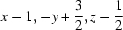
; (iii) 
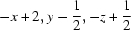
.

**Fig. 1.**
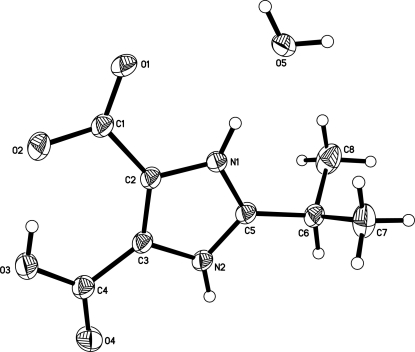
The title compound with displacement ellipsoids drawn at the 30% probability level.

**Fig. 2.**
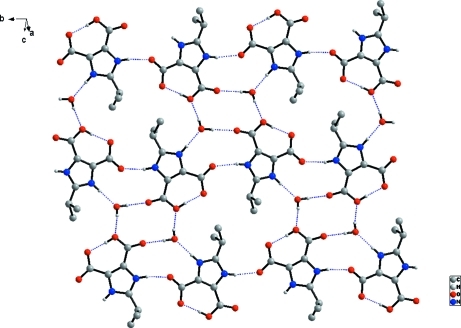
A view of the crystal structure. The H-atoms not included in hydrogen bonding have been omitted for clarity.

## supplementary crystallographic information

### Comment

In the past the construction of metal complexes based on N-heterocyclic carboxylic acids has attracted much attention due to their intriguing topologies as well as their potential applications in many fields. Particular attention has been paid to the 1*H*-imidazole-4,5-dicarboxylic acid ligand and its analogs: (Sun *et al.*, 2006) have synthesized the 4-carboxy-2-(pyridinium-4-yl)-1*H*-imidazole-5-carboxylate monohydrate; Merchan *et al.* (2007) have prepared the dimethylammonium 4-carboxy-1*H*-imidazole-5-carboxylate; (Guo, 2009) have reported the 4-carboxy-2-methyl-1*H*-imidazole-5-carboxylate monohydrate and (Wang & Qin, 2010) have reported the dimethylammonium 4-carboxy-2-n-propyl-1*H*-imidazole-5-carboxylate. All of these 1*H*-imidazole-4,5-dicarboxylic acid and their analogs have been used as ligands to design metal complexes and most of them are proved ideal ligands (Wang *et al.*, 2010; Feng *et al.*, 2010; Li *et al.*, 2010). However, the crystal structure of 4-carboxy-2-isopropyl-1*H*-imidazole-5-carboxylate has not been yet determined. Keeping that in mind, we report here the preparation and crystal structure of the title compound. The crystal structure (Fig.2, Table1) is stabilized by two intramolecular and three intermolecular N—H···O and O—H···O hydrogen bonds which link the molecules into  two-dimensional networks parallel to the (102) planes.

### Experimental

The title compound was synthesized according to the method reported in the literature (Alcalde *et al.*, 1992). Colourless single crystals suitable for X-ray diffraction were obtained by slow evaporation of a water solution of the compound.

### Refinement

H atoms bonded to the water O atom were located in an electron density map and refined with distance restraints of O—H = 0.85 Å. Other H atoms were positioned geometrically and refined using a riding model, with C—H = 0.96—0.98 Å, N—H = 0.86 Å and O—H = 0.82 Å. *U*_iso_(H) = k*U*_eq_(carrier atom), where k = 1.2 for N and C_tertiary_ and 1.5 for O and C_methyl_.

### Crystal data

**Table:**

C_8_H_10_N_2_O_4_·H_2_O	*F*(000) = 456
*M**_r_* = 216.20	*D*_x_ = 1.439 Mg m^−^^3^
Monoclinic, *P*2_1_/*c*	Mo *K*α radiation, λ = 0.71073 Å
Hall symbol: -P 2ybc	Cell parameters from 1379 reflections
*a* = 7.828 (2) Å	θ = 2.7–25.1°
*b* = 14.308 (4) Å	µ = 0.12 mm^−^^1^
*c* = 8.930 (2) Å	*T* = 298 K
β = 93.590 (3)°	Block, colourless
*V* = 998.2 (4) Å^3^	0.40 × 0.32 × 0.28 mm
*Z* = 4	

### Data collection

**Table:**

Bruker SMART APEXII CCD area-detector diffractometer	2147 independent reflections
Radiation source: fine-focus sealed tube	1599 reflections with *I* > 2σ(*I*)
graphite	*R*_int_ = 0.028
φ and ω scans	θ_max_ = 27.0°, θ_min_ = 2.6°
Absorption correction: multi-scan (*SADABS*; Bruker, 2005)	*h* = −9→9
*T*_min_ = 0.953, *T*_max_ = 0.967	*k* = −17→18
4874 measured reflections	*l* = −11→7

### Refinement

**Table:**

Refinement on *F*^2^	Secondary atom site location: difference Fourier map
Least-squares matrix: full	Hydrogen site location: inferred from neighbouring sites
*R*[*F*^2^ > 2σ(*F*^2^)] = 0.043	H-atom parameters constrained
*wR*(*F*^2^) = 0.123	*w* = 1/[σ^2^(*F*_o_^2^) + (0.059*P*)^2^ + 0.133*P*] where *P* = (*F*_o_^2^ + 2*F*_c_^2^)/3
*S* = 1.06	(Δ/σ)_max_ < 0.001
2147 reflections	Δρ_max_ = 0.32 e Å^−^^3^
140 parameters	Δρ_min_ = −0.19 e Å^−^^3^
3 restraints	Extinction correction: *SHELXTL* (Sheldrick, 2008), Fc^*^=kFc[1+0.001xFc^2^λ^3^/sin(2θ)]^-1/4^
Primary atom site location: structure-invariant direct methods	Extinction coefficient: 0.016 (3)

### Special details

**Table:**

Geometry. All esds (except the esd in the dihedral angle between two l.s. planes) are estimated using the full covariance matrix. The cell esds are taken into account individually in the estimation of esds in distances, angles and torsion angles; correlations between esds in cell parameters are only used when they are defined by crystal symmetry. An approximate (isotropic) treatment of cell esds is used for estimating esds involving l.s. planes.
Refinement. Refinement of F^2^ against ALL reflections. The weighted R-factor wR and goodness of fit S are based on F^2^, conventional R-factors R are based on F, with F set to zero for negative F^2^. The threshold expression ofF^2^ > 2sigma(F^2^) is used only for calculating R-factors(gt) etc. and is not relevant to the choice of reflections for refinement. R-factors based on F^2^ are statistically about twice as large as those based on F, and R-factors based on ALL data will be even larger.

### Fractional atomic coordinates and isotropic or equivalent isotropic displacement parameters (Å^2^)

**Table:**

	*x*	*y*	*z*	*U*_iso_*/*U*_eq_	
C4	1.2174 (2)	0.88114 (12)	0.4485 (2)	0.0397 (4)	
C2	1.0189 (2)	0.76673 (11)	0.29253 (18)	0.0346 (4)	
C3	1.0710 (2)	0.85223 (11)	0.34578 (19)	0.0350 (4)	
C6	0.6833 (2)	0.91545 (12)	0.1177 (2)	0.0413 (4)	
H6	0.6971	0.9835	0.1205	0.050*	
C1	1.0889 (2)	0.67085 (11)	0.3136 (2)	0.0391 (4)	
C5	0.8360 (2)	0.87217 (11)	0.19793 (19)	0.0366 (4)	
C7	0.5240 (2)	0.89011 (17)	0.1978 (2)	0.0593 (6)	
H7A	0.5139	0.8233	0.2031	0.089*	
H7B	0.4249	0.9154	0.1435	0.089*	
H7C	0.5328	0.9156	0.2975	0.089*	
C8	0.6644 (3)	0.88445 (16)	−0.0461 (2)	0.0622 (6)	
H8A	0.7620	0.9051	−0.0971	0.093*	
H8B	0.5624	0.9113	−0.0934	0.093*	
H8C	0.6571	0.8175	−0.0507	0.093*	
O2	1.21461 (18)	0.66102 (9)	0.41079 (17)	0.0588 (4)	
O4	1.24232 (16)	0.96417 (9)	0.47291 (15)	0.0527 (4)	
O3	1.31065 (17)	0.81470 (9)	0.50578 (16)	0.0521 (4)	
H3	1.2783	0.7647	0.4692	0.078*	
O1	1.02199 (16)	0.60794 (8)	0.23815 (16)	0.0494 (4)	
N1	0.87377 (17)	0.78132 (9)	0.20091 (15)	0.0368 (3)	
H1	0.8162	0.7384	0.1529	0.044*	
N2	0.95551 (16)	0.91583 (9)	0.28360 (16)	0.0372 (4)	
H2	0.9601	0.9752	0.2981	0.045*	
O5	0.66848 (18)	0.65027 (9)	0.07026 (18)	0.0638 (5)	
H5B	0.5604	0.6549	0.0562	0.096*	
H5A	0.6938	0.5927	0.0646	0.096*	

### Atomic displacement parameters (Å^2^)

**Table:**

	*U*^11^	*U*^22^	*U*^33^	*U*^12^	*U*^13^	*U*^23^
C4	0.0340 (9)	0.0334 (10)	0.0514 (10)	0.0000 (7)	0.0003 (7)	−0.0017 (8)
C2	0.0306 (8)	0.0264 (8)	0.0464 (9)	0.0013 (6)	−0.0014 (7)	0.0008 (7)
C3	0.0308 (8)	0.0258 (8)	0.0483 (10)	0.0020 (6)	0.0007 (7)	0.0004 (6)
C6	0.0366 (9)	0.0297 (9)	0.0565 (11)	0.0066 (7)	−0.0047 (7)	0.0023 (7)
C1	0.0346 (9)	0.0256 (9)	0.0566 (10)	0.0024 (7)	−0.0013 (7)	0.0012 (7)
C5	0.0336 (9)	0.0260 (8)	0.0499 (10)	0.0017 (7)	−0.0005 (7)	0.0001 (7)
C7	0.0405 (11)	0.0786 (16)	0.0581 (12)	0.0123 (10)	−0.0015 (9)	0.0096 (11)
C8	0.0637 (13)	0.0724 (16)	0.0502 (12)	0.0243 (12)	0.0007 (10)	0.0104 (10)
O2	0.0564 (9)	0.0348 (8)	0.0813 (10)	0.0108 (6)	−0.0258 (7)	0.0023 (6)
O4	0.0521 (8)	0.0331 (7)	0.0711 (9)	−0.0084 (6)	−0.0102 (6)	−0.0041 (6)
O3	0.0438 (8)	0.0372 (7)	0.0725 (9)	0.0015 (6)	−0.0178 (6)	−0.0031 (6)
O1	0.0450 (7)	0.0245 (6)	0.0775 (9)	0.0024 (5)	−0.0040 (6)	−0.0058 (6)
N1	0.0336 (7)	0.0242 (7)	0.0514 (8)	0.0017 (6)	−0.0070 (6)	−0.0022 (6)
N2	0.0334 (8)	0.0205 (7)	0.0570 (9)	0.0013 (5)	−0.0028 (6)	−0.0026 (6)
O5	0.0516 (8)	0.0357 (8)	0.0994 (11)	0.0046 (6)	−0.0323 (8)	−0.0154 (7)

### Geometric parameters (Å, °)

**Table:**

C4—O4	1.221 (2)	C5—N2	1.327 (2)
C4—O3	1.285 (2)	C5—N1	1.333 (2)
C4—C3	1.481 (2)	C7—H7A	0.9600
C2—C3	1.366 (2)	C7—H7B	0.9600
C2—N1	1.374 (2)	C7—H7C	0.9600
C2—C1	1.485 (2)	C8—H8A	0.9600
C3—N2	1.375 (2)	C8—H8B	0.9600
C6—C5	1.489 (2)	C8—H8C	0.9600
C6—C7	1.520 (3)	O3—H3	0.8200
C6—C8	1.526 (3)	N1—H1	0.8600
C6—H6	0.9800	N2—H2	0.8600
C1—O1	1.222 (2)	O5—H5B	0.8500
C1—O2	1.279 (2)	O5—H5A	0.8501
			
O4—C4—O3	124.60 (17)	C6—C7—H7A	109.5
O4—C4—C3	119.41 (16)	C6—C7—H7B	109.5
O3—C4—C3	115.99 (15)	H7A—C7—H7B	109.5
C3—C2—N1	106.79 (14)	C6—C7—H7C	109.5
C3—C2—C1	133.16 (16)	H7A—C7—H7C	109.5
N1—C2—C1	120.04 (15)	H7B—C7—H7C	109.5
C2—C3—N2	106.13 (14)	C6—C8—H8A	109.5
C2—C3—C4	131.93 (15)	C6—C8—H8B	109.5
N2—C3—C4	121.93 (14)	H8A—C8—H8B	109.5
C5—C6—C7	109.35 (15)	C6—C8—H8C	109.5
C5—C6—C8	111.52 (14)	H8A—C8—H8C	109.5
C7—C6—C8	110.41 (17)	H8B—C8—H8C	109.5
C5—C6—H6	108.5	C4—O3—H3	109.5
C7—C6—H6	108.5	C5—N1—C2	109.54 (14)
C8—C6—H6	108.5	C5—N1—H1	125.2
O1—C1—O2	125.32 (16)	C2—N1—H1	125.2
O1—C1—C2	117.95 (16)	C5—N2—C3	110.08 (14)
O2—C1—C2	116.73 (15)	C5—N2—H2	125.0
N2—C5—N1	107.44 (14)	C3—N2—H2	125.0
N2—C5—C6	126.67 (15)	H5B—O5—H5A	107.5
N1—C5—C6	125.84 (15)		
			
N1—C2—C3—N2	−0.31 (17)	C7—C6—C5—N2	−106.3 (2)
C1—C2—C3—N2	178.46 (17)	C8—C6—C5—N2	131.29 (19)
N1—C2—C3—C4	179.79 (16)	C7—C6—C5—N1	71.0 (2)
C1—C2—C3—C4	−1.4 (3)	C8—C6—C5—N1	−51.3 (2)
O4—C4—C3—C2	175.88 (18)	N2—C5—N1—C2	1.04 (18)
O3—C4—C3—C2	−3.9 (3)	C6—C5—N1—C2	−176.75 (15)
O4—C4—C3—N2	−4.0 (2)	C3—C2—N1—C5	−0.44 (18)
O3—C4—C3—N2	176.25 (16)	C1—C2—N1—C5	−179.40 (15)
C3—C2—C1—O1	−172.81 (18)	N1—C5—N2—C3	−1.24 (18)
N1—C2—C1—O1	5.8 (2)	C6—C5—N2—C3	176.52 (16)
C3—C2—C1—O2	7.7 (3)	C2—C3—N2—C5	0.97 (18)
N1—C2—C1—O2	−173.71 (15)	C4—C3—N2—C5	−179.12 (15)

### Hydrogen-bond geometry (Å, °)

**Table:**

*D*—H···*A*	*D*—H	H···*A*	*D*···*A*	*D*—H···*A*
O3—H3···O2	0.82	1.64	2.4576 (19)	175
N1—H1···O5	0.86	1.83	2.6879 (19)	171
N2—H2···O1^i^	0.86	1.93	2.7619 (19)	162
O5—H5B···O3^ii^	0.85	2.03	2.8684 (19)	171
O5—H5A···O4^iii^	0.85	1.94	2.7857 (19)	173

Symmetry codes: (i) −*x*+2, *y*+1/2, −*z*+1/2; (ii) *x*−1, −*y*+3/2, *z*−1/2; (iii) −*x*+2, *y*−1/2, −*z*+1/2.
